# Supplementary material for: Prognostic Value of Mid-Region Proadrenomedullin and In Vitro Interferon Gamma Production for In-Hospital Mortality in Patients with COVID-19 Pneumonia and Respiratory Failure: An Observational Prospective Study
Source: Viruses. 2022 Jul 30;14(8):1683. doi: 10.3390/v14081683 (PMC9414973; doi:10.3390/v14081683)

Supplementary materials:

Supplementary Table S1: clinical and laboratory characteristics of the study population other than what shown in Table 1

	**Overall (N= 100)**	**survivors (N= 87)**	**deceased (N= 13)**	**p value**
**COMORBIDITIES**				
Myocardial infarction	14 (14.0)	11 (12.6)	3 (23.1)	0.390
Congestive heart failure	3 (3.0)	0	3 (23.1)	**0.002**
Peripheral vascular disease	5 (5.0)	2 (2.3)	3 (23.1)	**0.015**
Cerebrovascular disease	7 (7.0)	6 (6.9)	1 (7.7)	1.000
Dementia	1 (1.0)	0	1 (7.7)	0.130
COPD	5 (5.0)	4 (4.6)	1 (7.7)	0.509
Connective tissue disease	0	0	0	-
Gastroduodenal ulcer	3 (3.0)	3 (3.5)	0	1.000
Hepatopathy	1 (1.0)	0	1 (7.7)	0.130
Diabetes mellitus	19 (19.0)	12 (13.8)	7 (53.8)	**0.003**
With organ damage	2 (2.0)	1 (1.2)	1 (7.7)	
Hemiplegia	1 (1.0)	1 (1.6)	0	1.000
Chronic renal failure (Moderate/severe)	4 (4.0)	3 (3.5)	1 (7.7)	0.432
Cancers	3 (3.0)	2 (2.3)	1 (7.7)	0.345
HIV infection in AIDS	0	0	0	-
**CLINICAL AND LABORATORY CHARACTERISTICS AT T0 (other than reported in Table 1)**				
SARS-Cov2 Variants/Lineages				0.401
Alpha (B.1.1.7)	30 (30.0)	24 (27.6)	6 (46.1)	
Delta (B.1.617.2)	24 (24.0)	22 (25.4)	2 (15.4)	
Eta (B.1.525)	1 (1.0)	1 (1.1)	0	
Gamma (P.1)	1 (1.0)	1 (1.1)	0	
Other lineages (no VOC detected)	44 (44.0)	39 (44.8)	5 (38.5)	
Leukocytes (mmc)	6.34 (4.78-8.82)	6.33 (4.76-8.97)	6.34 (5.42-7.83)	0.858
PCT (mcg/L) (n=49)	0.12 (0.07-0.23)	0.12 (0.06-0.24)	0.13 (0.10-0.23)	0.643
Ferritin (mcg/L)	791.0 (455.0-1332.0)	897.0 (470.0-1427.0)	520.0 (333.0-661.0)	**0.022**
**THERAPIES ADMINISTERED DURING HOSPITALIZATION**				
Antibiotic therapy	35 (35.0)	28 (32.2)	7 (53.9)	0.211
Remdesivir	25 (25.0)	25 (28.7)	0	**0.034**
Monoclonals casirivimab/imdevimab	3 (3.0)	3 (3.5)	0	1.000
**COMPLICATIONS OCCURRED DURING HOSPITALIZATION**				
Secondary infections	21 (21.0)	17 (19.5)	4 (30.8)	0.464
Thromboembolic events	9 (9.0)	8 (9.2)	1 (7.7)	1.000

Categorical variables are expressed as counts and percentages and continuous variables as medians and interquartile ranges. Legend: COPD chronic obstructive pulmonary disease, PCT procalcitonin

Supplementary Figure S1: AUROC analysis of currently employed inflammatory markers (lymphocyte count, CRP, ferritin levels) for in-hospital mortality, at T0 (**panel A**) and T1 (**panel B**).

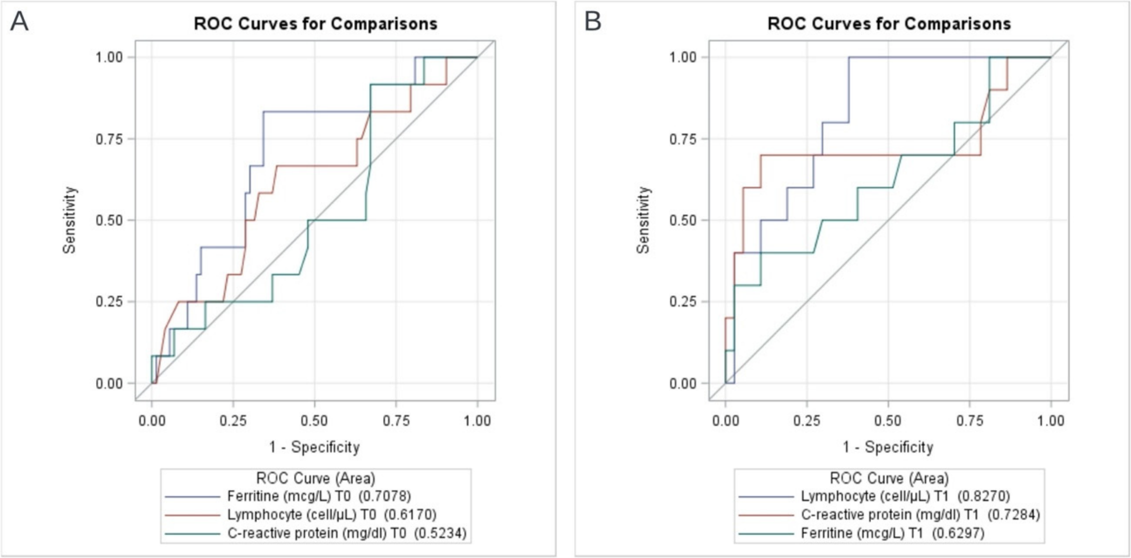

Supplement: Supplementary file 1 [file viruses-14-01683-s001.zip › viruses-1824422-supplementary.pdf]
